# From Postural Orthostatic Tachycardia Syndrome to Radiologically Isolated Syndrome

**DOI:** 10.1155/2018/2956387

**Published:** 2018-02-25

**Authors:** Richa Tripathi, Evanthia Bernitsas

**Affiliations:** ^1^Emory University School of Medicine, Atlanta, GA, USA; ^2^Wayne State School of Medicine, Detroit, MI, USA

## Abstract

**Background:**

Autonomic dysfunction is common in Multiple Sclerosis (MS) patients. Most spinal cord lesions entail some degree of autonomic nervous system dysfunction. MS patients may develop autonomic dysfunction later in their disease course.

**Methods:**

We report a patient with no prior history of MS presenting with orthostatic symptoms and diagnosed initially with postural orthostatic tachycardia syndrome (POTS). Four months later, she was diagnosed with radiologically isolated syndrome (RIS). The diagnosis was supported by imaging and CSF analysis.

**Conclusion:**

Our case sheds light on the need to consider autonomic dysfunction as an initial presentation of demyelinating pathology. Larger trials are needed to outline the possible association between POTS and RIS.

## 1. Introduction

Postural orthostatic tachycardia syndrome (POTS) is characterized by an orthostatic increase in the heart rate of ≥30 beats per minute within 10 minutes of standing or during head-up tilt table test in the absence of orthostatic hypotension. Another standard for the pediatric group (12–19 years of age) is an increment of 40 beats per minute for 12–19 years of age group. The symptoms are due to cerebral hypoperfusion and autonomic overactivity and are relieved in recumbent position. Symptoms include orthostatic lightheadedness, tachycardia, fatigue, and mental clouding. POTS can be associated with central nervous system (CNS) lesions including demyelinating diseases [1–3]. It was thought that most Multiple Sclerosis (MS) cases with autonomic dysfunction are advanced in their clinical course; however, recent literature supports the presence of POTS in clinically isolated syndrome [4]. Management of the symptoms in such patients is usually delicate as most medications have several side effects.

## 2. Case Presentation

Our patient is a 30-year old woman, previously healthy, who presented to the outpatient cardiology clinic for episodes of postural lightheadedness and subjective tremulousness of her whole body. Head-up tilt table test was consistent with POTS. The remaining workup was normal. She was maintained on metoprolol 25 mg twice a day and spironolactone 50 mg daily. Four months later, she presented to the neurology clinic for recurrent episodes of lightheadedness, intermittent neck pain, and nausea.

**Figure 1:**
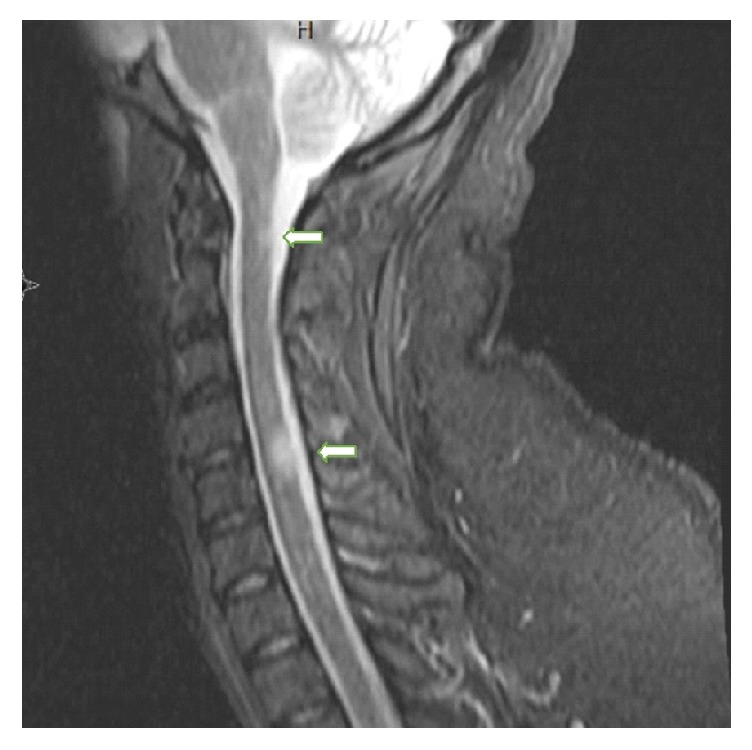
Sagittal T2-weighted images of the cervical spine showing abnormal signal cord at C2 and C4-5 level (arrows).

**Figure 2:**
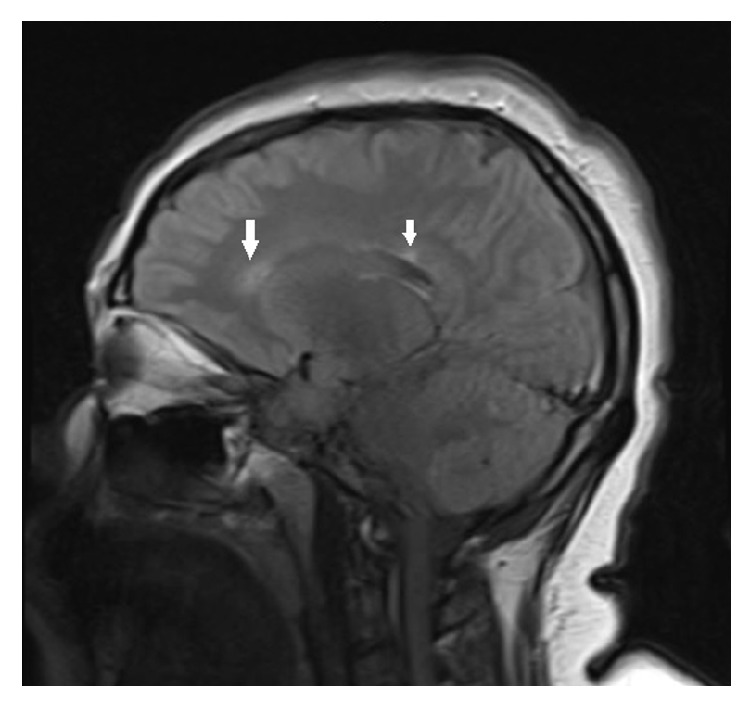
FLAIR sagittal images of the brain showing pericallosal hyperintensities. FLAIR: fluid- attenuated inversion recovery (arrows).

Her neurological examination was unremarkable. She underwent Magnetic Resonance Imaging (MRI) scan of her cervical spine without contrast for her neck pain. It demonstrated T2 hyperintensities at C2 and C4-5 level, suspicious for demyelination (Figure 1). We further requested MRI scan of the brain with contrast, which showed several periventricular and pericallosal hyperintensities (Figure 2).

Cerebrospinal fluid (CSF) analysis showed 16 nucleated cells (97% lymphocytes), high IgG index of 2.11 (normal limits 0–0.85), 21 CSF oligoclonal bands, and 1 serum oligoclonal band. Blood work including ANA, anti-dsDNA, ANNAT1, ANNAT2, and ANNAT3, PCCAB 1, 2, and 3, anti-Sm, anti-RNP, anti-SSA, anti-SSB, anti-Scl70, anti-Jo1, ANCA, amphiphysin, striated muscle antibody, and CRMP-5 was normal. Serum NMO-IgG antibody was negative.

She was diagnosed with radiologically isolated syndrome (RIS) and she was started on glatiramer acetate. On follow-up, she reported occasional neck pain and continued to experience lightheadedness. One year later, she is clinically stable and she is being followed up in the MS clinic.

## 3. Discussion

An extensive literature review suggests that most patients develop POTS either several years prior to being diagnosed with MS or as a part of autonomic dysfunction associated with MS [1]. Our literature search revealed only one such case with pure autonomic dysfunction in the form of POTS as a presenting symptom of MS reported by Kuba et al. [5].

In a large retrospective study, Adamec et al. [6] reported a significant association between POTS and MS. Several mechanisms have been proposed for the development of these symptoms including sympathetic denervation and central causes such as sympathetic overdrive, hypovolemia, and involvement of the renin-angiotensin-aldosterone system. Sympathetic nervous system (SNS) dysfunction is a direct consequence of spinal cord injury and is due to loss of supraspinal control. It has been reported that the clinical symptomatology of SNS dysfunction is more common with higher spinal cord pathology above the level of mid thoracic cord. Furthermore, the higher the level, the greater the degree of severity of SNS dysfunction [7].

The Mayo Clinic experience study showed some correlation between autoimmunity and POTS. This study entails specific antibodies, such as ganglionic acetylcholine receptor antibody [8] and alpha-1 adrenergic receptor antibody that act as partial antagonists and induce reflex compensatory sympathetic activation [9].

Our patient was diagnosed with RIS that has not converted to clinically isolated syndrome (CIS) or definite MS yet. Her age and the presence of spinal cord lesions predict a higher risk for conversion to CIS or MS in the next 5 years [10]. In a recently published prospective, observational study, Habek et al. [4] reported that POTS was present in 9.5% of patients with clinically isolated syndrome (CIS). In this study, age and POTS were found to predict early conversion from CIS to MS.

Up till now, there are no case reports or studies focused on a possible association between RIS and POTS, and the role of POTS in predicting conversion from RIS to CIS or definite MS is still unknown. Our case report highlights the need for further studies to explore the presence and significance of POTS in RIS.
